# Role of Autophagy in Cadmium-Induced Hepatotoxicity and Liver Diseases

**DOI:** 10.1155/2021/9564297

**Published:** 2021-08-10

**Authors:** Suryakant Niture, Minghui Lin, Qi Qi, John T. Moore, Keith E. Levine, Reshan A. Fernando, Deepak Kumar

**Affiliations:** ^1^Julius L. Chambers Biomedical Biotechnology Research Institute, North Carolina Central University, Durham, NC 27707, USA; ^2^The Fourth People's Hospital of Ningxia Hui Autonomous Region, Yinchuan 750021, China; ^3^RTI International, Research Triangle Park, Durham, NC 27709, USA

## Abstract

Cadmium (Cd) is a toxic pollutant that is associated with several severe human diseases. Cd can be easily absorbed in significant quantities from air contamination/industrial pollution, cigarette smoke, food, and water and primarily affects the liver, kidney, and lungs. Toxic effects of Cd include hepatotoxicity, nephrotoxicity, pulmonary toxicity, and the development of various human cancers. Cd is also involved in the development and progression of fatty liver diseases and hepatocellular carcinoma. Cd affects liver function via modulation of cell survival/proliferation, differentiation, and apoptosis. Moreover, Cd dysregulates hepatic autophagy, an endogenous catabolic process that detoxifies damaged cell organelles or dysfunctional cytosolic proteins through vacuole-mediated sequestration and lysosomal degradation. In this article, we review recent developments and findings regarding the role of Cd in the modulation of hepatotoxicity, autophagic function, and liver diseases at the molecular level.

## 1. Introduction

**Figure 1:**
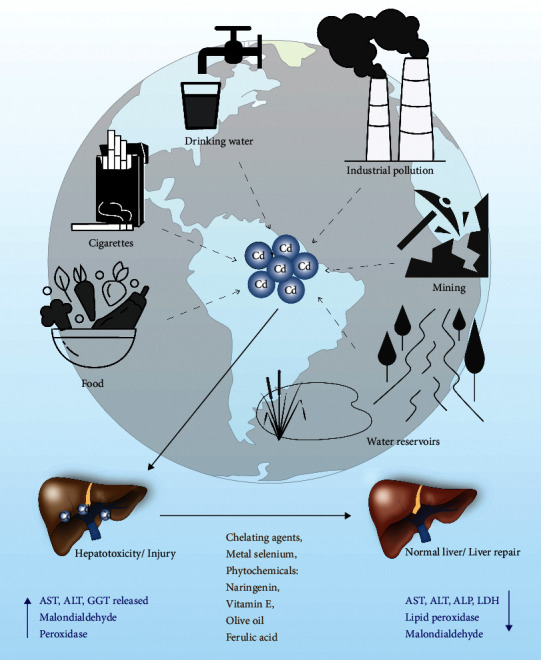
The schematic model represents the different environmental sources of Cd that impact liver toxicity. Cd accumulation increases liver toxicity and induces liver damage. Cd accumulation increases the expression of ALT, AST, GGT, MDA, and peroxidase activities, a prominent liver damage marker. On the other hand, pre- or postexposure with chelating agents, metals like Se and several phytochemicals could reverse the Cd-mediated hepatic toxicity/damage.

Cadmium (Cd) is a nonessential heavy metal in mammals; it can cause toxic damage to major tissues and organs and affects many physiological systems [1]. Cd is listed as a toxic substance endangering human health as reported by the Agency for Toxic Substances and Disease Registry (https://www.atsdr.cdc.gov/). The International Organization for Research on Cancer identified Cd and related compounds as the first category of carcinogens [2]. In developing countries, Cd pollution is becoming an increasingly serious problem and is adversely affecting human health [3, 4]. The main sources of Cd pollution include drinking water, contaminated food, cigarette smoking, mineral mining, smelting, industrial applications, and the use of Cd batteries (Figure 1) [5]. The prevalent modes of Cd intake by humans are inhalation, dermal contact, and ingestion through the air, soil, sediment, and water [6].

Apart from occupational exposure and personal tobacco consumption, diet accounts for about 90% of Cd exposure [7]. In some studies, the absorption rate of Cd intake through diet is estimated to be 3% to 5% in humans [8]; however, other studies suggest that absorption through diet can be as high as 44% [9]. Cd is an accumulative toxicant with a long biological half-life that has been estimated to be 10 to 33 years in humans, which results in bioaccumulation of Cd in various organs and thus increased overall body burden over time [7]. After absorption, Cd is distributed throughout the body by blood circulation after binding with albumin [10, 11]. Cd changes the binding position of metal ions such as calcium, iron, and zinc ions on membrane transport proteins to change the permeability of cell membranes and enhancing its entry into cells [12], thus compounding its role in kidney, liver, and testicular damage; osteoporosis; cardiovascular disease; neurological disease; and immune system disorders [12].

In this review, we focus on the impact of Cd exposure on hepatotoxicity and related liver diseases. We also discuss how Cd modulates liver diseases through the modulation of autophagy, a naturally occurring cell regulatory mechanism that eliminates and recycles damaged cell organelles and toxic components to maintain cellular homeostasis.

## 2. Acute and Chronic Cd Exposure and Liver Toxicity

**Figure 2:**
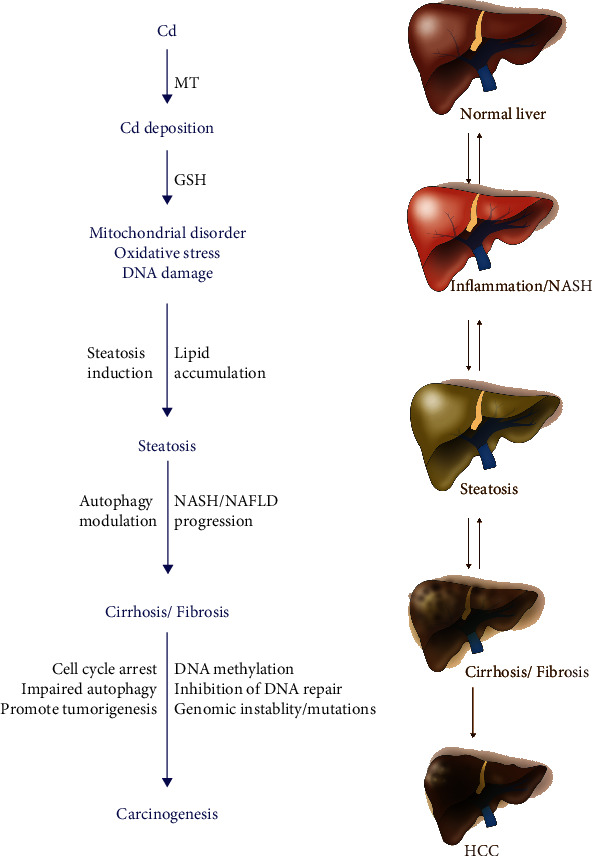
The schematic model represents the role of Cd in liver inflammation and the development of NASH, cirrhosis, and HCC. Cd deposition in the liver downregulates glutathione production and increases oxidative stress, mitochondrial dysfunction, and DNA damage. Cd deposition also enhances hepatic steatosis, which leads to an increase in severe liver lesions/damage and HCC progression.

The liver's main job is to filter the blood coming from the digestive tract before it circulates to the rest of the body, metabolizing and detoxifying potentially harmful chemicals and drugs. Approximately 60% of absorbed Cd is deposited in the liver (30%) and kidney (30%), while the rest is distributed throughout the other parts of the body [10]. Approximately 0.007% to 0.009% of the Cd body burden per day is excreted through urine and feces [10]. There are two main ways for the ionic form of Cd^2+^ to get through the hepatocyte cell membrane: (1) binding with Fe^2+^ and Zn^2+^ transporters, or (2) through voltage-gated Ca^2+^ channels [9, 13]. Protein-bound Cd usually binds to liver-produced metallothionein (MT) protein to form Cd metallothionein (Cd-MT) complex, which enters cells through receptor-mediated endocytosis [9, 13] (Figure 2) and is then released from the Cd-MT as a Cd ion through the digestion of lysosome. Chronic Cd ion is stored in various tissues such as the liver, kidney, prostate, and bone [14, 15]. In the female liver, Cd absorption is 10%–20% higher than in males, and the female liver is more susceptible to Cd toxicity. This difference may be related to progesterone-activated receptor-dependent calcium channels, channels that are involved in the absorption and accumulation of Cd into the liver [16]. This phenomenon may also be related to the lack of iron in the female body. Iron deficiency promotes the expression of divalent metal transporter 1 (DMT1) in cells, thereby enhancing the transport of divalent metal ions into the cells [17]. Deposition of Cd in the liver can cause both liver injury and hepatotoxicity [18].

Acute Cd poisoning causes increased levels of liver damage markers such as alanine aminotransferase (ALT), aspartate aminotransferase (AST), and alkaline phosphatase (ALP) in the blood and also increases the incidence of nonalcoholic hepatitis and fatty liver [19] (Figure 1). Histopathology shows that Cd can induce acute liver injury leading to tissue necrosis, apoptosis, hyperplasia, and enlarged liver sinus and hilum [20, 21]. Severe necrosis is accompanied by neutrophil infiltration and chemotaxis to the lesion [22], which can worsen liver damage. The mechanisms of Cd-induced acute hepatic injury mainly involve two pathways [23]. Firstly, Cd directly binds to sulfhydryl groups on key molecules including glutathione (GSH) and sulfhydryl groups of proteins to cause oxidative damage [23]. Secondly, although Cd is not a redox-reactive metal, it can cause inflammatory injury [23, 24], an event associated with oxidative stress [24] (Figure 2).

Cd is an indirect genotoxic carcinogen, and it has been shown that chronic Cd exposure strongly correlates with an increased risk of several cancers including prostate, genitourinary, breast, lung, pancreas, and hepatocellular carcinoma (HCC) [25–30]. Extensive research on the molecular mechanism of Cd carcinogenesis has shown that chronic Cd exposure can induce oxidative stress [31–33], inhibit DNA repair [34, 35], promote abnormal methylation of DNA [36–38], interfere with gene expression [29, 38], affect cell cycle regulation [39, 40], inhibit cell apoptosis [41], induce inflammatory signaling [42], and promote genomic instability and mutation in key genes to promote tumorigenesis [43]. Indeed, high concentrations of Cd from acute exposure or low concentrations of chronic exposure are both linked to severe hepatotoxicity/liver injury that promotes liver diseases and HCC development (Figure 2).

## 3. Cd Toxicity and Related Liver Diseases

Various cohort studies in the United States have revealed that urinary Cd levels are associated with the development of numerous cancers [44], cardiovascular disease [45], and increased mortality in men [46]. A cohort study in the nonsmoking population carried out in Tehran found a positive correlation coefficient (*r* = 0.66) between dietary and blood Cd, but not with dietary and urinary Cd. The study further suggests that increased Cd levels in the blood are linked to low serum ferritin levels [47]. Environmental exposure of Cd in humans can modulate innate and adaptive immunity, mucosal immune responses, chemokine release, increased susceptibility to pathogenic infections, and gene regulation [42]. Also, numerous metabolic factors increase the risk of fatty liver diseases, and a strong positive relationship was found between soil metal concentrations (Cr, Ni, Cu, and Cd) with fatty liver disease [48], risk of type 2 diabetes [49], and other metabolic morbidities [49].

Another cohort study further investigated an association between blood Cd, lead, and mercury levels with hepatic steatosis (HS) and fibrosis (HF) in human males and females. The study revealed that higher blood levels of heavy metals are positively correlated with hepatic steatosis index (HSI) and fibrosis-4 index (FIB-4) in females than males. Furthermore, blood mercury levels are associated with hepatic steatosis in males and females whereas higher blood Cd levels are positively correlated with hepatic fibrosis in females [50]. Furthermore, several metals including copper, zinc, nickel, and cobalt are detected at a lower concentration in serum from alcoholic liver cirrhosis patients; however, serum Cd concentration was significantly higher in patients with advanced alcoholic liver cirrhosis [51]. Similarly, Kazi et al. demonstrated that liver cirrhotic/cancer patients showed lower levels of Se and Zn in blood and serum, whereas at least twofold higher levels of As and Cd levels are found in serum and blood samples suggesting that Cd may increase the risk of severe liver diseases including HCC [52].

A strong relationship was observed between increased urinary Cd and increased serum levels of hepatic enzymes AST, ALT, and GGT, and exposure to Cd is associated with the development of hepatic nonalcoholic fatty liver disease (NAFLD), nonalcoholic steatohepatitis (NASH), and neuroinflammation in humans [53]. As part of its molecular mechanism, Cd modulates the function of both kidneys and liver by alteration of methylation of the *Klotho* gene. At lower concentrations, Cd exposure decreased *Klotho-*methylation whereas higher doses of Cd increased *Klotho-*methylation [54]. *Klotho-*methylation levels are positively correlated with the level of *β*_2_-microglobulin and negatively correlated with albumin levels, indicating Cd effects through this mechanism in both the liver and kidney [54]. Recently, Li et al. analyzed the histological changes that occur in the liver due to Cd exposure [55]. Raman confocal imaging demonstrated that liver tissue samples exposed to Cd showed decreased band intensity of cytochrome C at 748 cm^−1^, 1128 cm^−1^, and 1585 cm^−1^ and a higher collagen peak at 1082 cm^−1^, consistent with liver fibrosis [55]. Similarly, chronic Cd exposure in mice with 0.95 and 6.04 *μ*g/g w/w for 20 weeks induced NAFLD and NASH-like phenotypes, respectively. Exposure to Cd also suppresses the Sirtuin 1 (SIRT1) signaling pathway in the liver, resulting in hepatic mitochondrial dysfunction by inhibition of fatty acid oxidation [56].

In zebrafish, CdCl_2_ combined with human high-density lipoprotein (HDL) treatments induces the formation of multimeric apoAI and increased the production of the glycated form [57]. The study further showed that zebrafish fed with CdCl_2_ (12 and 24 *μ*M for 4 weeks) resulted in a dramatic increase in cholesterol and triglyceride (TG) levels, as well as markers of fatty liver, suggesting that CdCl_2_ induces hyperlipidemia and fatty liver changes by modulation of cholesteryl ester transfer protein (CETP) activity [57]. A dose-dependent feeding of hens with a Cd-containing diet for 9 weeks showed accumulation of Cd in the kidney, liver, pancreas, and lungs. Interestingly, a diet containing Cd at 30 mg Cd/kg induced the expression of antioxidant enzymes in the liver, whereas a diet containing Cd at 60 mg Cd/kg suppressed antioxidant enzyme activities [58]. Cd (60 mg/kg) increased reactive oxygen species (ROS), ER stress, apoptotic protein expression, bile duct hyperplasia, inflammatory cell infiltration, and periportal fibrosis in the liver by upregulation of cytokine TNF-*α*, IL-6, and IL-10 [58]. The diet containing 30 mg Cd/kg increased liver steatosis whereas Cd at high concentration (60 mg/kg) suppressed hepatic steatosis, suggesting that low-dose Cd may trigger defense mechanisms in the liver, whereas a higher concentration of Cd exposure may lead to liver injury in this model [58].

At the cellular level, exposure of hepatic cells with a low dose of Cd *in vitro* increased cell death-related pathways and enhanced JNK activation [59]. Exposure of HCC-derived Huh-7 cells with nanoparticles (10 nM) containing cadmium telluride quantum dots (CdTe QDs) induced cell death by increasing intracellular ROS, mitochondrial depolarization, and decreased DNA integrity in cells, suggesting that CdTe QDs can trigger cytotoxicity in liver cancer cells [60]. In studies of spontaneous transformation of cultured rat liver TRL 1215 cells, Cd regulated the methylation of apolipoprotein E (*ApoE*) gene, which is involved in the suppression of cell invasion. Cd treatments inhibit *ApoE* expression and reactivate *ApoE* expression when cells are treated with 5-aza-2′-deoxycytidine, a DNA demethylating agent, suggesting that Cd can induce promoter methylation of the *ApoE* gene. The study further pointed out that Cd enhances cell invasion because of the suppression of *ApoE* expression during the malignant transformation of TRL1215 liver cells [61]. Moreover, the same authors also pointed out that Cd-induced excessive promoter DNA methylation of *ApoE* results in the downregulation of *ApoE* gene expression leading to malignant transformation of TRL 1215 cells [62]. Similarly, when mice were exposed to a low concentration of Cd, alterations were observed in liver gene expression, lipid metabolism, mitochondrial oxidative phosphorylation, presence of liver enzymes in plasma, and fat accumulation in the liver, indicating that Cd may be involved in NAFLD/NASH development [59]. Recently, the effect of whole-life Cd exposure with a high-fat diet (HFD) in mice was analyzed [63]. Mouse offspring showed a significant accumulation of Cd in their kidney, liver, and heart, and female offspring showed increased accumulation of Cd in kidney and liver compared with males. Interestingly, mice fed with HFD containing Cd showed a twofold accumulation of Cd in the kidney, liver, and heart and also an increase in essential metal levels in blood, kidney, and liver compared with mice fed with a low-fat diet (LFD) and Cd. The study revealed that Cd may interact with HFD and affect essential metal homeostasis, which can promote the development of obesity-related diseases including fatty liver diseases [63]. In short, human cohort studies and animal studies confirm that Cd-induced liver toxicity modulates liver disease in animals and humans (Figure 2).

## 4. Cd Acute Hepatotoxicity Can Be Reversible

Recent reports highlight that several chelating agents [10], metals [64, 65], chemopreventive phytochemicals [66–69], and natural drugs [70] can reverse Cd-induced liver cytotoxicity and prevent liver damage (Figure 1). For example, the lipophilic chelating agent MiADMSA can promote the elimination of heavy metals (lead, cobalt, cadmium, manganese, iron, and copper) in the urine of hepatitis C virus (HCV) patients suggesting that chelation therapy could be useful to reverse heavy metal-related health problems [71]. Amino acid-L-carnitine (LC) and metal selenium (Se) also modulate Cd-induced liver toxicity in mice. Combined administration of LC and SeCl_4_ reduced Cd-mediated oxidative stress, Cd-mediated increase in ALT and AST levels, and lipid peroxidation in the liver and increased antioxidant enzymatic activities, suggesting that LC and SeCl_4_ can act synergistically to prevent Cd-mediated liver injury in mice [72]. A naturally occurring phytochemical-flavonoid catechin, catechin with phospholipid (CT-PH) complex, was shown to reduce Cd-mediated liver injury in rat liver [73]. The study suggests that the greater bioavailability of the CT-PH complex can increase its benefits on liver function by regulation of lipid peroxidation and by increasing antioxidant enzyme expression, suggesting that CT-PH may protect from Cd-induced liver injury in rats [73]. The citrus flavanone naringenin also prevents Cd-induced hepatic toxicity in the rat liver [74]. Administration of naringenin (50 mg/kg) decreased serum hepatic markers including ALT, ALP, AST, and lactate dehydrogenase (LDH) and decreased lipid peroxidation in the liver induced by Cd toxicity and oxidative damage (Figure 1). Naringenin also restored the levels of antioxidant defense to normal levels and preserved the normal histological architecture of the liver tissue [74] (Figure 1). Similarly, a protective effect of alpha-tocopherol (vitamin E) on Cd toxicity in rat liver has been shown. Coadministration of vitamin E (300 mg/Kg/day for 3 weeks) decreased prooxidative state hepatic markers such as malondialdehyde (MDA) and peroxidase (POD) activities that are induced by Cd exposure and also increased superoxide dismutase (SOD) and catalase (CAT) activities, restored Ca levels, and improved liver architecture [75]. In addition, olive oil and colocynth oil prevented oxidative damage in Wistar rat livers induced by Cd. Cotreatment with olive oil or colocynth oil restored the antioxidant potential in plasma and liver and decreased MDA levels and transaminase activity [76]. Ferulic acid (FA) derivatives of curcumin also contribute to liver repair. An interesting recent study demonstrated that supplementation of FA (50 mg/kg) significantly decreased Cd accumulation in rat liver and kidney tissues by elevating antioxidant enzyme expression and by decreasing the expression of hepatonephrotoxicity enzymes [77]. The metal selenium (Se) also shows a protective effect against Cd-induced toxicity in rat liver. Coadministration of Se and Cd significantly increased serum cytokines including IL-1*β*, IL-6, TNF-*α*, IL-10, MDA, and antioxidant (GSH, CAT, GPx, and SOD) enzyme activities and reversed Cd-induced liver and kidney damage [78]. Interestingly, Horiguchi et al. [17] demonstrated that overexpression of IL8 in transgenic mice (hIL-8 Tg) inhibited neutrophil migration into the liver and increased the severity of liver damage after acute administration of Cd [22] (Figure 2).

Taken together, these studies indicate that Cd-mediated acute liver injury may be repairable with cotreatments of chelating agents, chemopreventive compounds, and metals (Figure 1). However, restoring liver function by these agents depends on the amount of Cd accumulation in the liver and the severity of liver damage. The potential utility of Cd toxicity protective agents requires further attention.

## 5. Cd Alters Hepatic Gene Expression and Cellular Pathways

Evidence indicates that Cd accumulation in the liver modulates several cell signaling pathways and cellular processes resulting in liver damage. Zhang et al. [79] analyzed Cd-mediated differential gene expression and found that Cd regulates several genes including *EGR1*, *FOSL1*, *ITGA2*, *EDN1*, and *IER3,* which play an important role in liver cell proliferation and metabolism-related pathways. Moreover, another study showed that exposure to a low dose of Cd (rats exposed to 20 nmol/kg, every other day for 4 weeks) induced hypermethylation of several hepatic genes and led to increased proliferation in hepatic cells [80]. Using methylated DNA immunoprecipitation-CpG island microarray technology, Wang et al. [80] identified 675 hypermethylated genes, 899 hypomethylated genes, and 55 genes with mixed hyper- and hypomethylation. The authors suggested that hypermethylation and gene silencing of the caspase-8 (*CASP8*) and *TNF* genes were key factors leading to decreased cell death and increase hepatic cell proliferation [80]. Cd and tetrabromobisphenol A (TBBPA) contaminants from e-waste recycling facilities induced the expression of several genes that are involved in liver damage in mice. Cd/TBBPA mixture induces apoptosis-related and phase I detoxification enzyme expression whereas Cd/TBBPA mixture downregulates the expression of phase II detoxification enzymes and leads to excessive ROS production in the mouse liver [81]. Cd also alters liver metabolism by modulating liver metabolomics. Subchronic exposure of Cd in combination with the pesticide chlorpyrifos (CPF) impairs energy balance and amino acid/fatty acid metabolism in the rat liver [82]. CD-CPF mixture modulates 11 biomarkers in the liver including three metabolites (butanedioic acid, Myo-inositol, and urea). The study indicates that Cd may facilitate CPF metabolism because of an antagonistic interaction between Cd and CPF [82].

Because Cd increases the risk of HCC, identification of key genes that are differentially expressed after Cd exposure can lead to an increased understanding of HCC progression. Using microarray-based gene expression profiles, Zhang et al. [14] identified two key genes, *SLC7A11* and *ITGA2,* which are upregulated after Cd exposure. The study suggests that *SLC7A11* and *ITGA2* might be involved in liver cell damage/transformation or the development of HCC [14]. Gene expression profile after acute or chronic exposure of Cd was analyzed in HCC HepG2 cells, and the study showed that acute exposures of Cd altered the expression of 333 genes, whereas chronic exposure of Cd altered 181 genes [83]. Moreover, Cd increased the expression of metallothionein (MT) proteins, which are involved in detoxification, and decrease the expression of monooxygenase *CYP3A7*, which regulates drug and lipid metabolism. The study suggests that acute Cd exposure modulates several key genes that modulate HCC cell morphology, small molecule synthesis, lipid metabolism, organization, and development whereas chronic exposure of Cd regulates the expression of genes that are involved in regulating cell signaling, cell cycle, organ morphology, and cancer [83]. These experimental data suggest that Cd modulates gene expression that modulates multiple cellular pathways in the liver.

## 6. Role of Autophagy in Cd-Induced Hepatotoxicity and Liver Diseases

According to the ways in which intracellular material is transported into the lysosome, autophagy can be divided into three types: macro-, micro-, and chaperone-mediated autophagy (CMA). Since Cd is involved in hepatotoxicity and also modulates liver function in which autophagic function plays an important role, in this review, we will focus on macroautophagy (henceforth termed autophagy) because it is most relevant to Cd-induced hepatotoxicity.

Autophagy is an evolutionary conserved catabolic process that involves the transport of damaged organelles, misfolded proteins, and other toxic macromolecular substances to the lysosome for degradation [84] and thus maintains cellular homeostasis [85]. Autophagy is induced when cells encounter stress (such as starvation, oxidative stress, drug and metal toxicity, infection, anoxia, and DNA damage) in their environment. The molecules that result from degraded macromolecules, such as nucleosides and amino acids, can be used to generate energy for cell survival [86]. Moreover, autophagy reduces the accumulation of abnormal proteins and aging organelles by eliminating damaged or toxic harmful substances from cells, thereby maintaining the stability of the intracellular environment. In the process of autophagy, misfolded protein substrates are wrapped in a double-layered membrane structure forming autophagic vesicles with a diameter between 400 and 900 nm to form the autophagosome, and then the outer membrane of the autophagosome is fused with the lysosomal membrane to form the autolysosome [87, 88].

Dysregulation of autophagy is closely related to a wide range of physiological and pathological processes including aging [89], inflammatory response [90, 91], immune surveillance [90], and tumorigenesis [92, 93]. Dysregulation of autophagic function is strongly associated with the development of Alzheimer's disease and other neurodegenerative diseases [94], and a recent report indicates that autophagic function is responsible for liver pathogenic conditions that include NAFLD and AFLD and is also responsible for increasing hepatotoxicity mediated by viral infection, aflatoxin, or metals [94]. The role of autophagy in the modulation of liver disease [95] including HCC was reviewed recently [85, 95–98].

Autophagy is regulated and more than 30 autophagy-related genes (*ATGs*)/proteins have been characterized in yeast and mammals [99]. Five key processes are involved in the generation of the autophagy complex and degradation of substrate: (1) formation of the phagophore, (2) formation of Atg5-Atg12-Atg16L complex and fusion with autophagosome, (3) lipidation of microtubule-associated protein light chain3 (LC3-II) which combines with autophagic vesicles to form the autophagosomes, (4) capture by autophagosomes of proteins and organelles that need to be degraded or removed, and (5) combining of autophagosomes with lysosomes to form autolysosomes that degrade the cargo in the autolysosome [96]. In mammals, autophagosomes are formed by a lipid bilayer membrane derived from the endoplasmic reticulum or the trans-Golgi and endosomes [100–103]. Autophagosome formation is regulated by many proteins and their complexes, including mammalian rapamycin complex 1 (mTORcl), Unc-51-like kinase (ULKl) complex, two ubiquitin-like protein (Atg12 and Atg8/LC3) conjugation systems, and the Beclin-1/class III phosphatidylinositol 3-kinase (PI3K) complex [104, 105].

Autophagy is induced by ROS through inhibition of AKT/mTOR signaling [106, 107]. Autophagy can be induced by ROS through direct modification of key proteins involved in the autophagy process, including Atg4, Atg5, and Beclin-1, and indirect alteration of signaling pathway molecules including JNK and p38 [106, 107]. Activation of autophagy reduces oxidative damage and degrades cellular toxic components, thus supporting tumor cell survival and promoting cancer growth and metastasis [108, 109].

Earlier studies indicate that autophagy not only promotes tumor development but is also involved in tumor suppression [85,110]. In liver diseases, the activation of different signaling pathways can coordinate different stages of autophagy formation, which has multiple links to cell growth, proliferation, senescence, and apoptosis [85, 111, 112]. A recent study demonstrated that Atg7 knockdown in nutrient-starved HCC Hep3B cells inhibits autophagosome formation, whereas knockdown of Atg7 also decreased HCC cell proliferation and tumorigenesis in a murine HCC mouse model induced by activated RAS, suggesting that functional autophagy promotes HCC tumorigenesis [113]. In contrast, Di Fazio et al. showed that the pan-deacetylase inhibitor panobinostat, which is known to induce cell death by modulating endoplasmic reticulum stress, upregulated autophagic function by increasing expression of Beclin-1 and Map1LC3B in HepG2 xenografts in nude mice treated with panobinostat [114]. Stable expression of GFP-RFPtag Map1LC3B combined with panobinostat treatment induced autophagosome formation and maturation. Interestingly, the study further revealed that exposure to autophagy inducer tamoxifen and panobinostat induced HCC cell death [114]. This evidence suggests a dual role of autophagy in HCC. With this background, we review studies that analyze the role of Cd in the modulation of autophagic function and liver toxicity.

### 6.1. Cd Induces Autophagy in the Liver

Epidemiological and experimental studies suggest that Cd increases ROS, liver toxicity, and damage to liver cell organelles and even promotes carcinogenesis, whereas autophagy eliminates ROS and damaged cell organelles. Oxidative stress is a crucial factor in inducing hepatotoxicity during Cd exposure [115]. Kim et al. [57] showed that exposure to CdCl_2_ in zebrafish resulted in acceleration of histopathological fatty liver changes, elevated blood levels of the hepatic enzymes GOT and GPT, and increased production of ROS. The detailed mechanism of how Cd regulates autophagy was investigated by Meng et al. [116] using the human embryonic normal liver cell line (WRL-68). These studies showed Cd-induced autophagy by the upregulation of LC3B-II and mature cathepsin *L* and upregulation of autophagosome-lysosome fusion and through activation of lysosomal function which is associated with the production of lysosomal acid. The study further found that autophagosome-lysosome fusion depended on intracellular pH and intracellular Ca(2+) stores or regulation of Ca^2+^ channels/pumps [116]. Evidence from other laboratories indicates that several upstream signaling pathways and molecules are involved in Cd-mediated autophagy induction in liver cells and HCC. In an earlier study, Vergilio and de Melo [117] analyzed the role of Cd in the regulation of autophagy, apoptosis, and cell organelle features after exposure of Cd to HCC cells Huh-7. Their study suggests that Cd exposure decreased HCC cell survival and induced morphological changes including cytoplasm retraction, nuclear condensation, loss of cell adhesion, dysfunctional mitochondria and ER, and acidified cytoplasm indicating cell death via apoptotic and autophagic pathway regulation [117]. The role of autophagy and gap junctional intercellular communication (GJIC) in Cd-induced apoptosis was analyzed by monitoring apoptotic nuclear morphological changes and cell index (CI) counting in rat liver cells [118]. Cd significantly inhibits GJIC, connexin 43 (Cx43), and reduced CI, whereas 6 h Cd treatment increased autophagy by enhancing the expression of autophagy regulatory proteins such as Atg5, Atg7, Beclin-1, and LC-II and protected the cells from Cd-induced apoptosis. The study also found that autophagy modulated Cd-induced GJIC inhibition that affects cellular fate [118]. Recently, the effect of coexposure of lead (Pb) and Cd on the regulation of autophagy in rat liver was also investigated [119]. Exposure of Pb, Cd, or a combination of Cd plus Pb increased the expression of autophagy-related protein mRNAs such as *ATG5*, *ATG7*, *Beclin-1*, *p62*, and *LC3* and induced autophagosome formation in rat livers [119].

Because selenium (Se) metal mitigates the Cd effect [65, 120], the effect of Se on Cd-mediated autophagy and cell apoptosis was analyzed in primary hepatocytes and the data showed that Se induced the expression of cytoprotective transcription factor Nrf2, which prevented Cd-mediated induction of autophagy and cell apoptosis by induction antioxidant enzyme expression [121]. Moreover, Cd exposure in avian leghorn male hepatoma (LMH) cells induced cell death by impairing intracellular Ca^2+^ homeostasis by inducing ER stress and autophagy [122]. This study also showed that treatment with Se induced Ca^2+^ homeostasis by activation of the Ca^2+^/calmodulin (CaM)/calmodulin kinase IV (CaMK-IV) signaling pathway, leading to Cd-mediated inhibition of ER stress and autophagy, suggesting that Cd-mediated induction of autophagy is controlled by CaMK-IV in LMH cells [122]. In contrast, Cd selenide (CdSe) quantum dots (QDs) induce hepatotoxicity that can be reversed by sulforaphane. Sulforaphane activates Nrf2 and upregulates the expression of antioxidant enzymes and induces autophagy that leads to decreased CdSe-mediated hepatotoxicity in mice [123]. Interestingly, the pineal gland hormone melatonin also prevents Cd toxicity in HCC cells [124]. Cd-induced mitochondrial-derived superoxide anion-dependent autophagic cell death by decreasing SIRT3 activity and protein expression and by enhancing the acetylation of SOD2 (mitochondrial superoxide dismutase 2) without disturbing the interaction between SIRT3 and SOD2 [124]. On the other hand, melatonin treatment increased SIRT1 activity, decreased the acetylation of SOD2, and inhibited autophagy induced by Cd *in vitro* and *in vivo,* indicating that melatonin exerts its hepatoprotective effect against Cd by decreasing autophagic cell death and by the regulation of SIRT3/SOD2 pathway [124]. Moreover, Cd caused an overactivation of mitophagy (mitochondria degradation) in normal human liver L02 cells, which depends on dynamin 1-like (DNM1L) expression [125]. Mechanistically, Cd induces mitophagy by inducing LC3-II expression and lysosomal colocalization with mitochondria, leading to an increase in the bioenergetic deficit in mitochondria and mitochondrial fragmentation. Cd also increases *DNM1L* expression and mitochondrial translocation. The study further suggests that inhibition of DNM1L by Mdivi-1 blocks abnormal mitophagy which leads to reduced Cd-induced hepatotoxicity *in vivo*, indicating that DNM1L and mitophagy signaling may play an important role in Cd-induced hepatotoxicity [125]. In short, these studies indicate a low level of Cd-induced functional autophagy for the reduction of hepatotoxicity.

## 7. Cd-Induced Defective Autophagy in the Liver Promotes Liver Diseases

**Figure 3:**
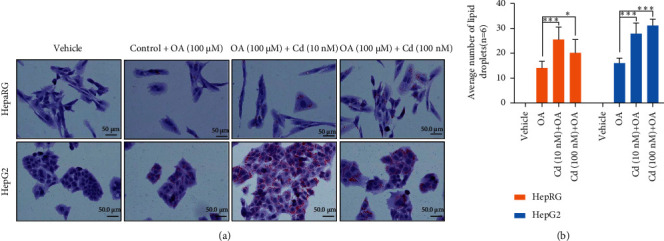
Cd increases HCC cell steatosis. (a) Normal liver HepaRG cells and HCC HepG2 cells were exposed with 10 nM and 100 nM of Cd for 30 h and cells were treated with oleic acid (100 *μ*M) for an additional 24 h. Cells were fixed with paraformaldehyde and stained with oil red O and images were captured and presented. (b) The effect of Cd on oleic acid-mediated lipid droplet formation was quantified and plotted. ^*∗*^*P* < 0.05, ^∗∗∗^*P* < 0.001 compared with oleic acid- (OA-) treated cells.

**Figure 4:**
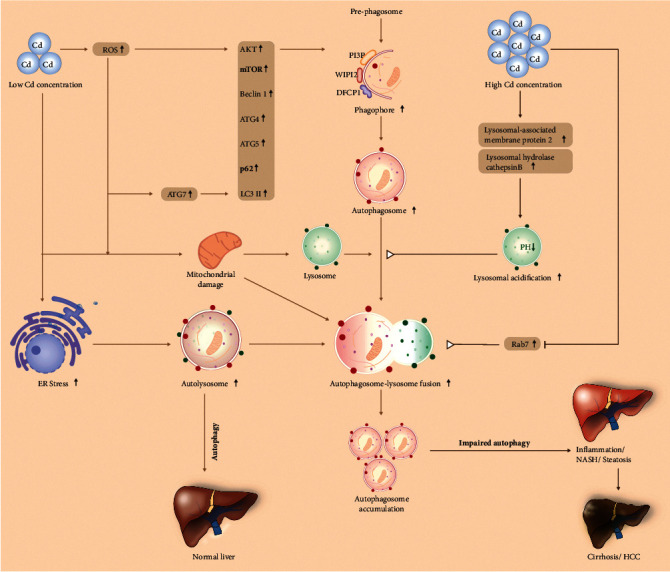
A schematic model represents a dual role of Cd in autophagy regulation in the liver. Low concentrations of Cd exposure increase ER and oxidative stress in the liver and the expression of autophagy regulatory components such as ATG7, ATG4, p62, LC3B, and Beclin-1, leading to increased function autophagy and reduced Cd-mediated liver toxicity. In contrast, at higher concentrations, there is Cd-induced defective autophagy by increasing lysosomal acidification and by blocking autophagosome-lysosome fusion, leading to increased liver steatosis, NAFLD, and HCC development.

In NAFLD, metabolic factors contribute to hepatic steatosis and autophagy. Rosales-Cru et al. [116] analyzed the effect of low cadmium acute treatment on hepatocytes obtained from mice fed with a high cholesterol diet. A study suggests that Cd treatment increases steatosis/hyperlipidemia in hepatocytes fed with cholesterol due to the induction of defective autophagy that is unable to degrade lipid contents [126]. In our laboratory, we also analyzed the effect of Cd-mediated cell steatosis in nontransformed HepaRG liver cells and HCC-derived HepG2 cells, and our data showed that exposure of both cell types to Cd resulted in increased cell steatosis (Figures 3(a) and 3(b)). Similar observations were also noted in mouse liver cells, where Cd was found to play a dual role in the induction of autophagy and/or impaired autophagy [127] (Figure 4). The study revealed that exposure to Cd increased lysosomal acidification both *in vivo* and *in vitro* by induction of lysosomal-associated membrane protein 2 and lysosomal hydrolase cathepsin B and enhanced the lysosomal degradation capacity. However, Cd inhibits Rab7 protein expression leading to impaired fusion of autophagosomes with lysosomes and thus increased hepatotoxicity [127]. Puerarin (PU), a potent free radical scavenger, is known to protect hepatocytes from Cd-induced cell death [128]. A recent study suggests that PU prevents Cd-induced hepatotoxicity, decreased Cd-mediated ROS and malondialdehyde production, and suppressed cell apoptosis. Mechanistically, Cd blocks autophagic flux in AML-12 cells, increases autophagosome accumulation, and induces impaired autophagy. However, posttreatment with PU alleviates autophagosome accumulation and restored normal autophagy in mouse hepatocytes suggesting that PU reduces Cd-mediated hepatic cell damage [128].

Overall, these studies indicate that activation of normal autophagy may be helpful to reduce Cd-mediated hepatotoxicity, whereas defective/impaired autophagy induced by Cd contributes to increasing the risk of liver diseases including HCC.

## 8. Conclusions and Future Perspectives

In this review, we summarize the important role of Cd in the induction of liver diseases. Environmental pollution is a big concern in developing countries and the presence of Cd in water, air, and the industrial sector has become a major problem because Cd targets the liver, lung, and kidney and causes serious disease lesions. This review focuses on Cd-mediated hepatotoxicity and numerous reports suggest that Cd is involved in the development and progression of NAFLD and HCC. Several human cohort studies and animal studies reveal that the higher presence of Cd in blood or serum is strongly associated with liver injury and NAFLD development. Cd not only induces hepatotoxicity but also affects liver function by modulation of cellular signaling pathways and cellular processes. The timing of Cd exposure and its mode of action is important during liver disease development. Cd regulates liver fibrosis and cirrhosis and is indirectly involved in HCC formation. Interestingly, Cd regulates autophagic function, and autophagy either prevents Cd-mediated hepatotoxicity by detoxifying damaged cellular organelles/molecules or promotes Cd-mediated liver diseases. The dual role of autophagy in Cd-mediated hepatotoxicity adds more complexity to the role of autophagy in Cd toxicity and may point to the importance of differentiating the effects of Cd under conditions of normal autophagy versus impaired autophagy. Studies have shown that normal autophagic function protects hepatocytes from Cd toxicity, whereas defective autophagic function results in more severe hepatic lesions. Cd-mediated defective autophagic function occurs because of a lack of fusion of autolysosome with the lysosome and therefore autophagy is unable to recycle damaged molecules. However, it is not clear at what stage of NAFLD or HCC that Cd induces defective autophagy. To control impaired autophagy and related risk of Cd, monitoring and identification of appropriate methods to assess autophagic flux (by analyzing the regulation of autophagy-related genes/protein markers) are important next steps. To normalize autophagy in the early stages of HCC or NAFLD diseases, new strategies such as vector-based expression of autophagy regulatory proteins in early disease, or the development of specific drugs that target autophagy, are required to reduce Cd toxicity [129, 130]. Moreover, chelating agents, metals like Se, and chemopreventive phytochemicals also reverse Cd toxicity by upregulation of antioxidant defense mechanisms, by normalizing the autophagic process, or by neutralizing Cd toxic effects. Because Cd directly accumulates in the liver, the identification of optimal methods to employ such molecules in the clinic could be an effective strategy to control Cd-mediated hepatotoxicity in early disease and should be the subject of increased research.

## Data Availability

All data presented in this study are included in this article and will be available from the corresponding author on request.

## Abbreviations

Cd: Cadmium
HCC: Hepatocellular carcinoma
MT: Metallothionein
Cd-MT: Cd metallothionein
DMT1: Divalent metal transporter 1
ALT: Alanine aminotransferase
AST: Aspartate aminotransferase
ALP: Alkaline phosphatase
GSH: Glutathione
T2DM: Type 2 diabetes mellitus
HS: Hepatic steatosis
HF: Hepatic fibrosis
HSI: Hepatic steatosis index
FIB-4: Fibrosis-4 index
GGT: Gamma-glutamyl transferase
NAFLD: Nonalcoholic fatty liver disease
NASH: Nonalcoholic steatohepatitis
SIRT1: Sirtuin 1
HDL: High-density lipoprotein
TC: Cholesterol
TG: Triglyceride
CETP: Cholesteryl ester transfer protein
ROS: Reactive oxygen species
ER: Endoplasmic reticulum
TNF-*α*: Tumor necrosis factor *α*
IL-6: Interleukin-6
HFD: High-fat diet
LFD: Low-fat diet
CdTe QDs: Cadmium telluride quantum dots
ApoE: Apolipoprotein E
HCV: Hepatitis C virus
LC: L-Carnitine
Se: Selenium
CT-PH: Phytochemical-flavonoid catechin or catechin with phospholipid
LDH: Lactate dehydrogenase
MDA: Lactate dehydrogenase
POD: Peroxidase
SOD: Superoxide dismutase
CAT: Catalase
FA: Ferulic acid
TBBPA: Tetrabromobisphenol A
CPF: Chlorpyrifos
CMA: Chaperone-mediated autophagy
LAMP-2A: Lysosomal-associated membrane protein 2A
ATGs: Autophagy-related genes
mTORcl: Mammalian rapamycin complex 1
ULKl: Unc-51-like kinase
PI3K: Beclin-1/class III phosphatidylinositol 3-kinase
GJIC: Gap junctional intercellular communication
CI: Cell index
Pb: Lead
LMH: Leghorn male hepatoma
CaM: Ca^2+^/calmodulin
CaMK-IV: Calmodulin kinase IV
CdSe: Cadmium selenide
QDs: Quantum dots
SOD2: Superoxide dismutase 2
DNM1L: Dynamin 1-like
Rab7: Ras-related protein Rab7
PU: Puerarin.
